# 1-(4-Chloro-3-fluoro­phen­yl)-2-[(3-phenyl­isoquinolin-1-yl)sulfan­yl]ethanone

**DOI:** 10.1107/S1600536809001573

**Published:** 2009-01-17

**Authors:** P. Manivel, Venkatesha R. Hathwar, T. Maiyalagan, N. Burcu Arslan, F. Nawaz Khan

**Affiliations:** a Chemistry Division, School of Science and Humanities, VIT University, Vellore 632 014, Tamil Nadu, India; b Solid State and Structural Chemistry Unit, Indian Institute of Science, Bangalore 560 012, Karnataka, India; c Ondokuz Mayıs University, Arts and Sciences Faculty, Department of Physics, 55139-Samsun, Turkey

## Abstract

In the title compound, C_23_H_15_ClFNOS, the isoquinoline system and the 4-chloro-3-fluoro­phenyl ring are aligned at 80.4 (1)°. The dihedral angle between the isoquinoline system and the pendant (unsubstituted) phenyl ring is 19.91 (1)°.

## Related literature

For related structures, see: Hathwar *et al.* (2008 ▶); Manivel *et al.* (2009*a* ▶,*b* ▶).
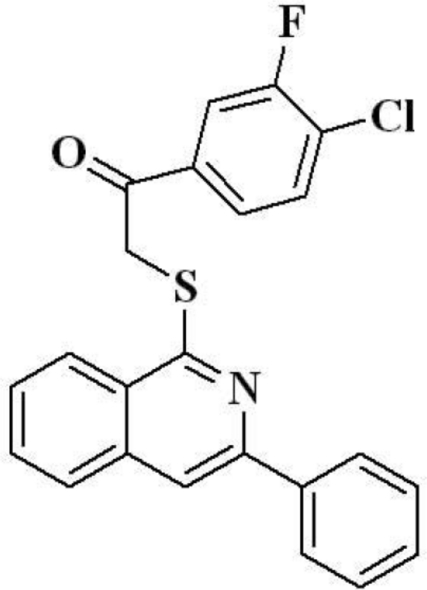


## Experimental

### 

#### Crystal data

C_23_H_15_ClFNOS
*M* *_r_* = 407.87
Orthorhombic, 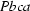

*a* = 16.9008 (11) Å
*b* = 9.8036 (7) Å
*c* = 23.3226 (16) Å
*V* = 3864.3 (5) Å^3^
*Z* = 8
Mo *K*α radiation
μ = 0.33 mm^−1^
*T* = 290 (2) K
0.24 × 0.18 × 0.11 mm

#### Data collection

Bruker SMART CCD area-detector diffractometer
Absorption correction: multi-scan (*SADABS*; Sheldrick, 1996 ▶) *T*_min_ = 0.925, *T*_max_ = 0.965
27428 measured reflections
3595 independent reflections
2424 reflections with *I* > 2σ(*I*)
*R*_int_ = 0.063

#### Refinement

*R*[*F* ^2^ > 2σ(*F* ^2^)] = 0.056
*wR*(*F* ^2^) = 0.128
*S* = 1.04
3595 reflections
253 parameters
H-atom parameters constrained
Δρ_max_ = 0.32 e Å^−3^
Δρ_min_ = −0.19 e Å^−3^

### 

Data collection: *SMART* (Bruker, 2004 ▶); cell refinement: *SAINT* (Bruker, 2004 ▶); data reduction: *SAINT*; program(s) used to solve structure: *SHELXS97* (Sheldrick, 2008 ▶); program(s) used to refine structure: *SHELXL97* (Sheldrick, 2008 ▶); molecular graphics: *ORTEP-3* (Farrugia,1997 ▶) and *CAMERON* (Watkin *et al.*, 1993 ▶); software used to prepare material for publication: *PLATON* (Spek, 2003 ▶).

## Supplementary Material

Crystal structure: contains datablocks global, I. DOI: 10.1107/S1600536809001573/ng2534sup1.cif

Structure factors: contains datablocks I. DOI: 10.1107/S1600536809001573/ng2534Isup2.hkl

Additional supplementary materials:  crystallographic information; 3D view; checkCIF report

## Figures and Tables

**Fig. 1.**
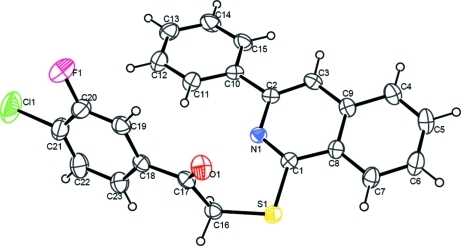
ORTEP diagram of the asymmetric unit of (I) with 50% probability displacement ellipsoids.

**Fig. 2.**
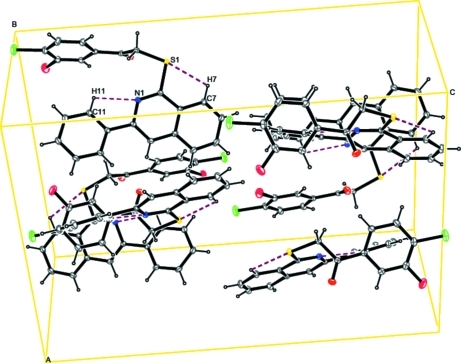
The crystal packing diagram of (I).The dotted lines indicate intermolecular C—H···O hydrogen bonds. All H atoms have been omitted for clarity.

## supplementary crystallographic information

### Comment

In compound (I), the S atom also located in the plane. The F atom deviates by 0.014 A from mean plane of phenyl ring containing F and Cl atoms. In this ring F– C and Cl—C bond distances are 1.348 (4) A, 1.727 (3) A, respectively. The orientation of isoquinoline ring system with respect to the another phenyl ring is given by the torsion angles for N1—C2—C10—C15 and C3—C2—C10—C11 are respectively -160.1 (2)°, -163.1 (3)° similarly for C16—S1—C1—N1 and C16—S1—C1—C8 are respectively -0.8 (2)° and 179.56 (19)° (Table 1).

### Experimental

3-Phenylisoquinoline-1-thiol and 2-bromo-1-(3-fluoro-4-chlorophenyl)ethanone were mixed in the ratio 1:1.05 equivalents with ethanol in a round bottom flask. Then it was heated under nitrogen atmosphere on an oil bath at 323 K. After 2 h, the products were filtered and dissolved in chloroform. Further, it was washed with water, dried and concentrated. The single-crystal for X-ray structue anlaysis was obtained from ether solution by slow evaporation.

### Refinement

All the H atoms in (I) were positioned geometrically and refined using a riding model with C—H bond lenghts of 0.93 Å and 0.97 Å for aromatic and for methylene H atoms respectively and *U*_iso_(H) = 1.2*U*_eq_(C) for all carbon bound H atoms.

### Crystal data

**Table:**

C_23_H_15_ClFNOS	*F*(000) = 1680
*M**_r_* = 407.87	*D*_x_ = 1.402 Mg m^−^^3^
Orthorhombic, *P**b**c**a*	Mo *K*α radiation, λ = 0.71073 Å
Hall symbol: -P 2ac 2ab	Cell parameters from 3595 reflections
*a* = 16.9008 (11) Å	θ = 1.8–25.5°
*b* = 9.8036 (7) Å	µ = 0.33 mm^−^^1^
*c* = 23.3226 (16) Å	*T* = 290 K
*V* = 3864.3 (5) Å^3^	Block, colourless
*Z* = 8	0.24 × 0.18 × 0.11 mm

### Data collection

**Table:**

Bruker SMART CCD area-detector diffractometer	3595 independent reflections
Radiation source: fine-focus sealed tube	2424 reflections with *I* > 2σ(*I*)
graphite	*R*_int_ = 0.063
φ and ω scans	θ_max_ = 25.5°, θ_min_ = 1.8°
Absorption correction: multi-scan (*SADABS*; Sheldrick, 1996)	*h* = −18→20
*T*_min_ = 0.925, *T*_max_ = 0.965	*k* = −11→11
27428 measured reflections	*l* = −28→28

### Refinement

**Table:**

Refinement on *F*^2^	Primary atom site location: structure-invariant direct methods
Least-squares matrix: full	Secondary atom site location: difference Fourier map
*R*[*F*^2^ > 2σ(*F*^2^)] = 0.056	Hydrogen site location: inferred from neighbouring sites
*wR*(*F*^2^) = 0.128	H-atom parameters constrained
*S* = 1.04	*w* = 1/[σ^2^(*F*_o_^2^) + (0.0566*P*)^2^ + 1.1665*P*] where *P* = (*F*_o_^2^ + 2*F*_c_^2^)/3
3595 reflections	(Δ/σ)_max_ = 0.001
253 parameters	Δρ_max_ = 0.32 e Å^−^^3^
0 restraints	Δρ_min_ = −0.19 e Å^−^^3^

### Special details

**Table:**

Geometry. All e.s.d.'s (except the e.s.d. in the dihedral angle between two l.s. planes) are estimated using the full covariance matrix. The cell e.s.d.'s are taken into account individually in the estimation of e.s.d.'s in distances, angles and torsion angles; correlations between e.s.d.'s in cell parameters are only used when they are defined by crystal symmetry. An approximate (isotropic) treatment of cell e.s.d.'s is used for estimating e.s.d.'s involving l.s. planes.

### Fractional atomic coordinates and isotropic or equivalent isotropic displacement parameters (Å^2^)

**Table:**

	*x*	*y*	*z*	*U*_iso_*/*U*_eq_	
O1	0.07142 (12)	0.8183 (2)	0.28005 (9)	0.0633 (6)	
F1	0.09785 (13)	0.8393 (2)	0.06905 (9)	0.1057 (7)	
S1	0.03138 (4)	0.61721 (7)	0.37120 (3)	0.0495 (2)	
Cl1	−0.02952 (8)	0.70329 (14)	0.00961 (4)	0.1221 (5)	
N1	0.12270 (12)	0.4853 (2)	0.29715 (9)	0.0399 (5)	
C1	0.11497 (15)	0.5201 (2)	0.35092 (11)	0.0393 (6)	
C2	0.18572 (14)	0.4054 (2)	0.28104 (11)	0.0405 (6)	
C3	0.24276 (16)	0.3684 (3)	0.31906 (11)	0.0474 (7)	
H3	0.2861	0.3180	0.3066	0.057*	
C4	0.29398 (18)	0.3701 (3)	0.41884 (13)	0.0593 (8)	
H4	0.3384	0.3205	0.4079	0.071*	
C5	0.2845 (2)	0.4076 (3)	0.47468 (14)	0.0688 (9)	
H5	0.3225	0.3827	0.5016	0.083*	
C6	0.2188 (2)	0.4828 (3)	0.49197 (13)	0.0643 (9)	
H6	0.2135	0.5085	0.5302	0.077*	
C7	0.16226 (18)	0.5189 (3)	0.45299 (12)	0.0538 (7)	
H7	0.1180	0.5676	0.4650	0.065*	
C8	0.17027 (16)	0.4832 (2)	0.39492 (11)	0.0422 (6)	
C9	0.23657 (16)	0.4063 (3)	0.37753 (11)	0.0451 (6)	
C10	0.18550 (14)	0.3638 (2)	0.21970 (11)	0.0419 (6)	
C11	0.13897 (17)	0.4309 (3)	0.18011 (12)	0.0515 (7)	
H11	0.1085	0.5046	0.1921	0.062*	
C12	0.13641 (19)	0.3920 (3)	0.12340 (12)	0.0597 (8)	
H12	0.1046	0.4392	0.0976	0.072*	
C13	0.18110 (19)	0.2828 (3)	0.10499 (13)	0.0641 (9)	
H13	0.1805	0.2569	0.0666	0.077*	
C14	0.22641 (19)	0.2130 (4)	0.14375 (14)	0.0715 (10)	
H14	0.2556	0.1379	0.1317	0.086*	
C15	0.22938 (17)	0.2524 (3)	0.20044 (13)	0.0598 (8)	
H15	0.2609	0.2042	0.2261	0.072*	
C16	−0.01524 (15)	0.6351 (3)	0.30300 (11)	0.0439 (6)	
H16A	−0.0177	0.5461	0.2849	0.053*	
H16B	−0.0692	0.6659	0.3089	0.053*	
C17	0.02538 (15)	0.7327 (2)	0.26264 (12)	0.0426 (6)	
C18	0.00791 (15)	0.7226 (2)	0.20013 (12)	0.0422 (6)	
C19	0.05916 (17)	0.7874 (3)	0.16253 (13)	0.0520 (7)	
H19	0.1020	0.8366	0.1766	0.062*	
C20	0.0465 (2)	0.7788 (3)	0.10523 (15)	0.0647 (9)	
C21	−0.0164 (2)	0.7098 (4)	0.08298 (14)	0.0679 (9)	
C22	−0.0680 (2)	0.6464 (3)	0.11953 (15)	0.0714 (9)	
H22	−0.1116	0.6000	0.1049	0.086*	
C23	−0.05585 (18)	0.6510 (3)	0.17826 (13)	0.0572 (8)	
H23	−0.0904	0.6062	0.2029	0.069*	

### Atomic displacement parameters (Å^2^)

**Table:**

	*U*^11^	*U*^22^	*U*^33^	*U*^12^	*U*^13^	*U*^23^
O1	0.0674 (14)	0.0532 (12)	0.0694 (14)	−0.0142 (11)	−0.0162 (11)	−0.0004 (10)
F1	0.1066 (17)	0.1363 (19)	0.0742 (14)	−0.0033 (15)	0.0267 (12)	0.0272 (13)
S1	0.0554 (5)	0.0517 (4)	0.0412 (4)	0.0142 (3)	0.0010 (3)	−0.0073 (3)
Cl1	0.1660 (12)	0.1504 (11)	0.0500 (6)	0.0166 (9)	−0.0195 (6)	0.0006 (6)
N1	0.0408 (13)	0.0394 (12)	0.0396 (12)	0.0018 (10)	0.0016 (10)	−0.0030 (9)
C1	0.0454 (16)	0.0322 (13)	0.0404 (15)	−0.0012 (11)	0.0021 (12)	−0.0003 (11)
C2	0.0372 (15)	0.0380 (14)	0.0462 (15)	−0.0010 (11)	0.0047 (12)	−0.0020 (12)
C3	0.0381 (15)	0.0473 (15)	0.0570 (17)	0.0062 (12)	0.0002 (14)	−0.0042 (14)
C4	0.0579 (19)	0.0559 (18)	0.064 (2)	0.0096 (15)	−0.0138 (16)	0.0034 (16)
C5	0.081 (2)	0.068 (2)	0.058 (2)	0.0080 (19)	−0.0261 (18)	0.0111 (17)
C6	0.090 (2)	0.0590 (19)	0.0443 (17)	0.0110 (18)	−0.0115 (17)	0.0026 (14)
C7	0.070 (2)	0.0462 (16)	0.0448 (17)	0.0078 (15)	−0.0052 (14)	0.0015 (13)
C8	0.0501 (16)	0.0346 (13)	0.0419 (15)	−0.0011 (12)	−0.0037 (12)	0.0025 (11)
C9	0.0481 (16)	0.0368 (14)	0.0505 (16)	−0.0013 (12)	−0.0068 (13)	0.0021 (12)
C10	0.0368 (15)	0.0425 (14)	0.0464 (16)	−0.0034 (12)	0.0067 (12)	−0.0055 (12)
C11	0.0665 (19)	0.0400 (15)	0.0479 (17)	0.0057 (14)	0.0020 (15)	−0.0024 (13)
C12	0.077 (2)	0.0564 (17)	0.0457 (17)	0.0053 (16)	−0.0019 (15)	0.0004 (14)
C13	0.065 (2)	0.081 (2)	0.0468 (18)	0.0032 (18)	0.0075 (16)	−0.0157 (16)
C14	0.058 (2)	0.091 (3)	0.065 (2)	0.0256 (19)	0.0001 (17)	−0.0299 (19)
C15	0.0466 (18)	0.074 (2)	0.0585 (19)	0.0217 (16)	−0.0017 (14)	−0.0164 (16)
C16	0.0429 (16)	0.0415 (15)	0.0472 (16)	0.0076 (12)	0.0010 (12)	−0.0031 (12)
C17	0.0383 (15)	0.0353 (14)	0.0543 (17)	0.0055 (12)	−0.0039 (13)	−0.0023 (12)
C18	0.0410 (15)	0.0341 (13)	0.0514 (17)	0.0041 (12)	−0.0020 (13)	0.0016 (12)
C19	0.0485 (17)	0.0474 (17)	0.060 (2)	0.0057 (13)	0.0024 (14)	0.0034 (14)
C20	0.069 (2)	0.069 (2)	0.056 (2)	0.0130 (18)	0.0129 (18)	0.0134 (16)
C21	0.087 (3)	0.072 (2)	0.0453 (18)	0.018 (2)	−0.0032 (18)	0.0037 (16)
C22	0.081 (2)	0.070 (2)	0.064 (2)	−0.0032 (18)	−0.0258 (19)	−0.0049 (17)
C23	0.0612 (19)	0.0512 (17)	0.059 (2)	−0.0033 (14)	−0.0071 (16)	0.0041 (14)

### Geometric parameters (Å, °)

**Table:**

O1—C17	1.215 (3)	C10—C15	1.395 (3)
F1—C20	1.348 (4)	C11—C12	1.377 (4)
S1—C1	1.768 (3)	C11—H11	0.9300
S1—C16	1.784 (3)	C12—C13	1.378 (4)
Cl1—C21	1.727 (3)	C12—H12	0.9300
N1—C1	1.306 (3)	C13—C14	1.368 (4)
N1—C2	1.375 (3)	C13—H13	0.9300
C1—C8	1.434 (3)	C14—C15	1.378 (4)
C2—C3	1.359 (3)	C14—H14	0.9300
C2—C10	1.487 (3)	C15—H15	0.9300
C3—C9	1.417 (3)	C16—C17	1.508 (4)
C3—H3	0.9300	C16—H16A	0.9700
C4—C5	1.363 (4)	C16—H16B	0.9700
C4—C9	1.413 (4)	C17—C18	1.491 (4)
C4—H4	0.9300	C18—C23	1.383 (4)
C5—C6	1.392 (4)	C18—C19	1.387 (4)
C5—H5	0.9300	C19—C20	1.356 (4)
C6—C7	1.366 (4)	C19—H19	0.9300
C6—H6	0.9300	C20—C21	1.363 (5)
C7—C8	1.405 (4)	C21—C22	1.369 (5)
C7—H7	0.9300	C22—C23	1.386 (4)
C8—C9	1.410 (3)	C22—H22	0.9300
C10—C11	1.380 (4)	C23—H23	0.9300
			
C1—S1—C16	99.63 (12)	C13—C12—H12	120.1
C1—N1—C2	119.3 (2)	C14—C13—C12	119.3 (3)
N1—C1—C8	123.8 (2)	C14—C13—H13	120.3
N1—C1—S1	118.51 (19)	C12—C13—H13	120.3
C8—C1—S1	117.71 (19)	C13—C14—C15	120.9 (3)
C3—C2—N1	121.5 (2)	C13—C14—H14	119.5
C3—C2—C10	123.8 (2)	C15—C14—H14	119.5
N1—C2—C10	114.7 (2)	C14—C15—C10	120.6 (3)
C2—C3—C9	120.4 (2)	C14—C15—H15	119.7
C2—C3—H3	119.8	C10—C15—H15	119.7
C9—C3—H3	119.8	C17—C16—S1	114.70 (19)
C5—C4—C9	120.2 (3)	C17—C16—H16A	108.6
C5—C4—H4	119.9	S1—C16—H16A	108.6
C9—C4—H4	119.9	C17—C16—H16B	108.6
C4—C5—C6	120.9 (3)	S1—C16—H16B	108.6
C4—C5—H5	119.6	H16A—C16—H16B	107.6
C6—C5—H5	119.6	O1—C17—C18	120.0 (2)
C7—C6—C5	120.2 (3)	O1—C17—C16	121.5 (3)
C7—C6—H6	119.9	C18—C17—C16	118.6 (2)
C5—C6—H6	119.9	C23—C18—C19	119.1 (3)
C6—C7—C8	120.6 (3)	C23—C18—C17	123.3 (3)
C6—C7—H7	119.7	C19—C18—C17	117.7 (2)
C8—C7—H7	119.7	C20—C19—C18	119.7 (3)
C7—C8—C9	119.2 (2)	C20—C19—H19	120.1
C7—C8—C1	124.3 (2)	C18—C19—H19	120.1
C9—C8—C1	116.5 (2)	F1—C20—C19	119.2 (3)
C8—C9—C4	118.9 (3)	F1—C20—C21	118.8 (3)
C8—C9—C3	118.4 (2)	C19—C20—C21	122.0 (3)
C4—C9—C3	122.7 (3)	C20—C21—C22	119.0 (3)
C11—C10—C15	117.4 (2)	C20—C21—Cl1	119.7 (3)
C11—C10—C2	120.9 (2)	C22—C21—Cl1	121.2 (3)
C15—C10—C2	121.6 (2)	C21—C22—C23	120.4 (3)
C12—C11—C10	121.9 (3)	C21—C22—H22	119.8
C12—C11—H11	119.0	C23—C22—H22	119.8
C10—C11—H11	119.0	C18—C23—C22	119.8 (3)
C11—C12—C13	119.8 (3)	C18—C23—H23	120.1
C11—C12—H12	120.1	C22—C23—H23	120.1
			
C2—N1—C1—C8	2.0 (4)	C15—C10—C11—C12	−1.1 (4)
C2—N1—C1—S1	−177.53 (17)	C2—C10—C11—C12	−178.4 (3)
C16—S1—C1—N1	−0.8 (2)	C10—C11—C12—C13	0.2 (4)
C16—S1—C1—C8	179.56 (19)	C11—C12—C13—C14	1.2 (5)
C1—N1—C2—C3	−4.0 (4)	C12—C13—C14—C15	−1.6 (5)
C1—N1—C2—C10	175.8 (2)	C13—C14—C15—C10	0.6 (5)
N1—C2—C3—C9	3.1 (4)	C11—C10—C15—C14	0.7 (4)
C10—C2—C3—C9	−176.7 (2)	C2—C10—C15—C14	178.0 (3)
C9—C4—C5—C6	−0.5 (5)	C1—S1—C16—C17	−73.20 (19)
C4—C5—C6—C7	0.7 (5)	S1—C16—C17—O1	−19.3 (3)
C5—C6—C7—C8	−1.2 (4)	S1—C16—C17—C18	160.73 (18)
C6—C7—C8—C9	1.6 (4)	O1—C17—C18—C23	−164.7 (3)
C6—C7—C8—C1	−178.2 (3)	C16—C17—C18—C23	15.2 (4)
N1—C1—C8—C7	−179.4 (2)	O1—C17—C18—C19	16.0 (4)
S1—C1—C8—C7	0.1 (3)	C16—C17—C18—C19	−164.1 (2)
N1—C1—C8—C9	0.7 (4)	C23—C18—C19—C20	−0.6 (4)
S1—C1—C8—C9	−179.71 (18)	C17—C18—C19—C20	178.7 (2)
C7—C8—C9—C4	−1.5 (4)	C18—C19—C20—F1	−178.3 (2)
C1—C8—C9—C4	178.4 (2)	C18—C19—C20—C21	1.2 (5)
C7—C8—C9—C3	178.5 (2)	F1—C20—C21—C22	179.0 (3)
C1—C8—C9—C3	−1.6 (3)	C19—C20—C21—C22	−0.5 (5)
C5—C4—C9—C8	0.9 (4)	F1—C20—C21—Cl1	−0.8 (4)
C5—C4—C9—C3	−179.1 (3)	C19—C20—C21—Cl1	179.7 (2)
C2—C3—C9—C8	−0.2 (4)	C20—C21—C22—C23	−0.8 (5)
C2—C3—C9—C4	179.8 (3)	Cl1—C21—C22—C23	179.0 (2)
C3—C2—C10—C11	−163.1 (3)	C19—C18—C23—C22	−0.7 (4)
N1—C2—C10—C11	17.1 (3)	C17—C18—C23—C22	−180.0 (3)
C3—C2—C10—C15	19.7 (4)	C21—C22—C23—C18	1.4 (5)
N1—C2—C10—C15	−160.1 (2)		

### Hydrogen-bond geometry (Å, °)

**Table:**

*D*—H···*A*	*D*—H	H···*A*	*D*···*A*	*D*—H···*A*
C7—H7···S1	0.93	2.68	3.076 (3)	107
C11—H11···N1	0.93	2.47	2.795 (4)	101

## References

[bb1] Bruker (2004). *SMART* and *SAINT* Bruker AXS Inc., Madison, Wisconsin, USA.

[bb2] Farrugia, L. J. (1997). *J. Appl. Cryst.* **30**, 565.

[bb3] Hathwar, V. R., Prabakaran, K., Subashini, R., Manivel, P. & Khan, F. N. (2008). *Acta Cryst.* E**64**, o2295. 10.1107/S160053680803609X PMC2959858 21581273

[bb4] Manivel, P., Hathwar, V. R., Nithya, P., Prabakaran, K. & Khan, F. N. (2009a). *Acta Cryst.* E**65**, o137–o138. 10.1107/S1600536808042062 PMC2968055 21581597

[bb5] Manivel, P., Hathwar, V. R., Nithya, P., Subashini, R. & Nawaz Khan, F. (2009*b*). *Acta Cryst.* E**65**, o254. 10.1107/S1600536809000257 PMC2968208 21581870

[bb6] Sheldrick, G. M. (1996). *SADABS* University of Göttingen, Germany.

[bb7] Sheldrick, G. M. (2008). *Acta Cryst.* A**64**, 112–122. 10.1107/S0108767307043930 18156677

[bb9] Spek, A. L. (2003). *J. Appl. Cryst.* **36**, 7–13.

[bb8] Watkin, D. J., Pearce, L. & Prout, C. K. (1993). *CAMERON* Chemical Crystallography Laboratory, University of Oxford, England.

